# Supplementary material for: Evaluation of Overall Survival by Restricted Mean Survival Time of Advanced Biliary Tract Cancer treated with Immunotherapy: A Systematic Review and Meta-Analysis
Source: Cancers (Basel). 2024 May 30;16(11):2077. doi: 10.3390/cancers16112077 (PMC11171222; doi:10.3390/cancers16112077)
Supplement: Supplementary file 1 [file cancers-16-02077-s001.zip › Figure Cancer_Suple.pptx]

## Slide 1
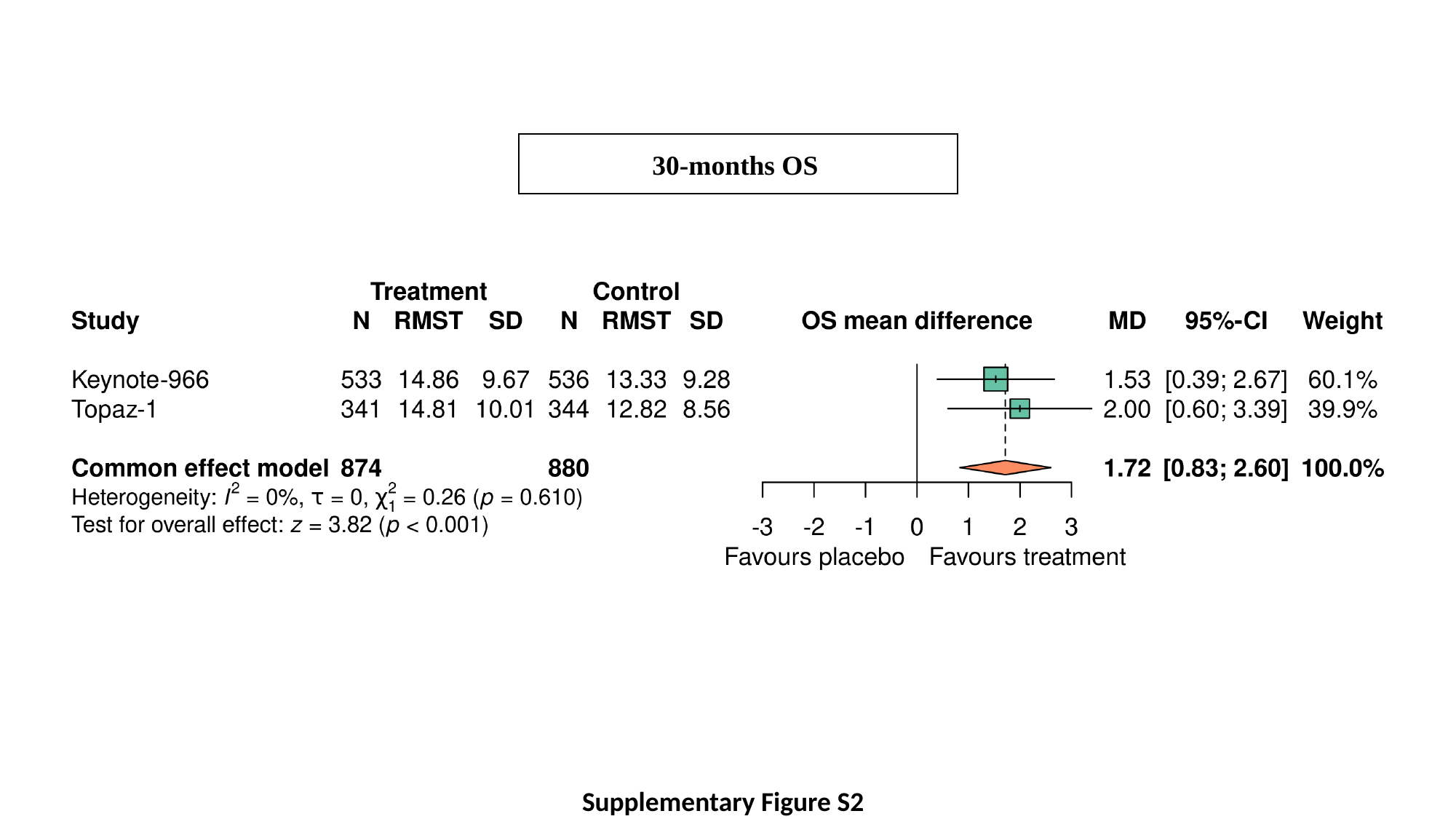

30-months OS
Supplementary Figure S2

## Slide 2
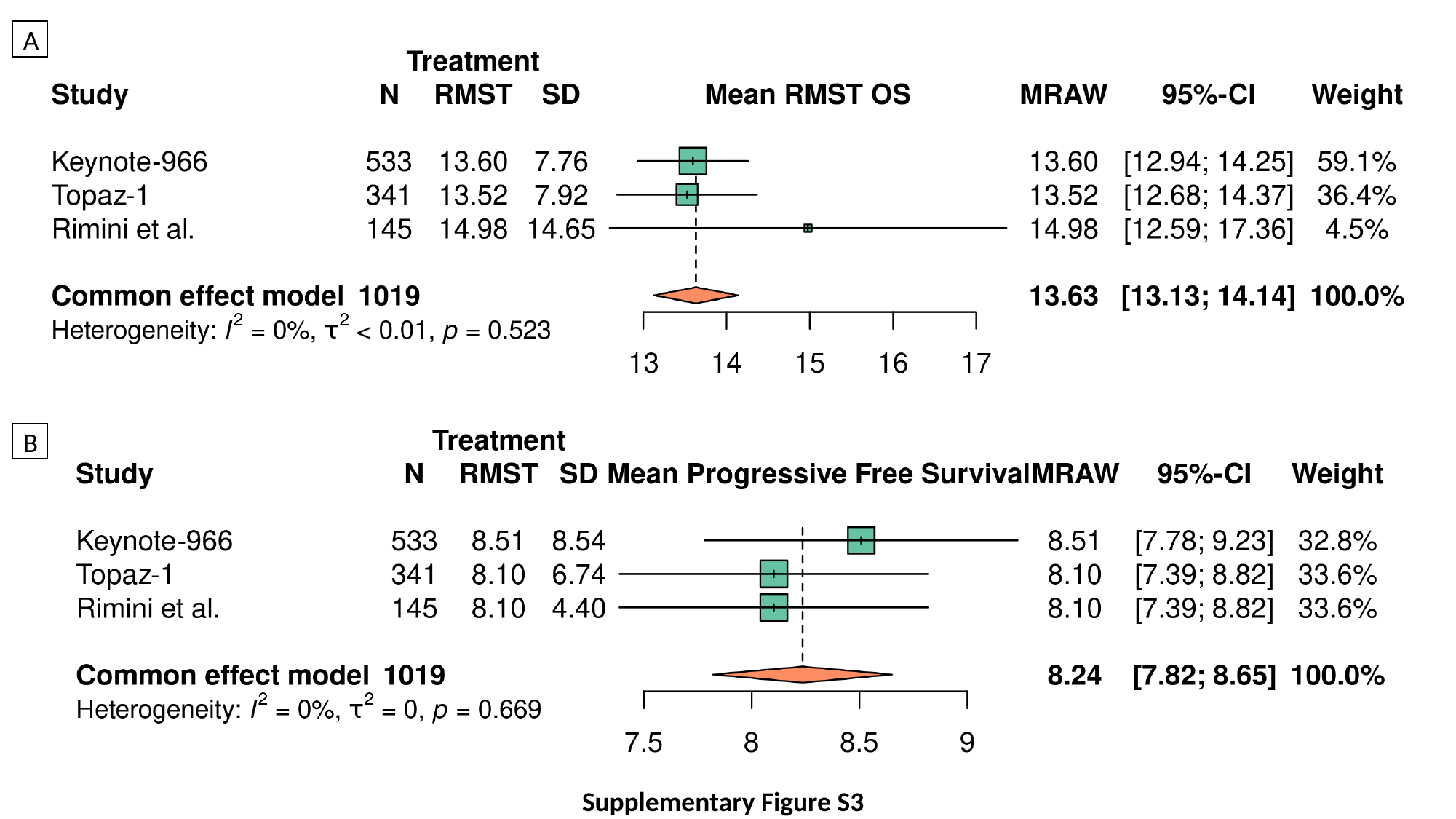

A
B
Supplementary Figure S3

## Slide 3
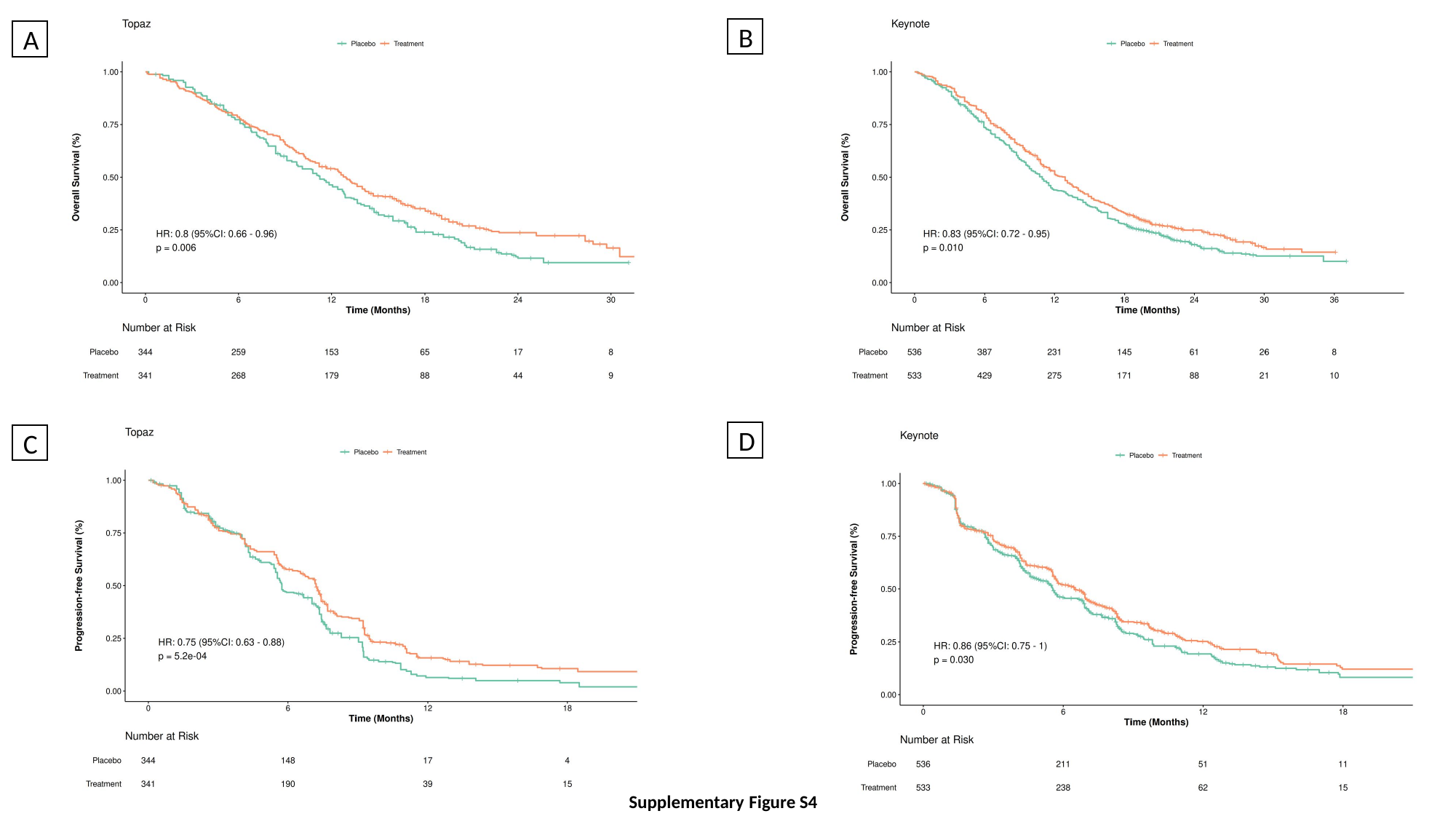

B
A
D
C
Supplementary Figure S4
