# Supplementary material for: Evaluation of Overall Survival by Restricted Mean Survival Time of Advanced Biliary Tract Cancer treated with Immunotherapy: A Systematic Review and Meta-Analysis
Source: Cancers (Basel). 2024 May 30;16(11):2077. doi: 10.3390/cancers16112077 (PMC11171222; doi:10.3390/cancers16112077)

Supplemental Digital Content

SEARCH STRATEGIES

Database	Platform	Date searched	Number of results
Medline	Ovid	11-08-2023	561
Embase	Elsevier	11-08-2023	1460
CENTRAL	Wiley	11-08-2023	201
Web of Science	FECYT	11-08-2023	84
**TOTAL WITH DUPLICATES**			2306
**TOTAL WITHOUT DUPLICATES**			1987

*Methodological filters for ECAs based on: https://www.sign.ac.uk/what-we-do/methodology/search-filters/

Ovid MEDLINE(R) ALL <1946 to November 8, 2023>

1 exp Biliary Tract Neoplasms/ 33908
2 ("biliary tract" adj3 (cancer? or tumo?r or neoplasm?)).ab,ti. 2949
3 ("bile duct" adj3 (cancer? or tumo?r or neoplasm?)).ab,ti. 2923
4 exp Cholangiocarcinoma/ 12536
5 cholangiocarcinoma?.ab,ti. 17976
6 (gallbladder adj3 (cancer? or tumo?r or neoplasm?)).ab,ti. 5880
7 (ampullary adj3 (cancer? or tumo?r or neoplasm?)).ab,ti. 888
8 (ampulla adj4 vater adj4 cancer).ab,ti. 205
9 or/1-8 45210
10 exp Immunotherapy/ 337359
11 Immuno*.ab,ti. 2151872
12 Immunization.ab,ti. 105252
13 "adoptive cell therap*".ab,ti. 1489
14 exp Immunosuppressive Agents/346260
15 (Immunosupressive adj2 (therap* or treatment?)).ab,ti. 108
16 Antibodies, Monoclonal/201501

17	"monoclonal antibod* ".ab,ti.	205357
18	exp Immune Checkpoint Inhibitors/	18720
19	"immune checkpoint inhibitor?".ab,ti.	22350
20	ICIs.ab,ti.	7135
21	PD-1.ab,ti.	27000
22	PD-L1.ab,ti.	25712
23	Nivolumab/	5351
24	opdivo.ab,ti.	79
25	nivolumab.ab,ti.	8529
26	camrelizumab.ab,ti.	527
27	pembrolizumab.ab,ti.	7942
28	durvalumab.ab,ti.	1428
29	Receptors, Immunologic/	26636
30	Immunologic Factors/	28776
31	Antineoplastic Agents, Immunological/	10481
32	(immunologic adj2 (factor? or receptor?)).ab,ti.	1216
33	or/10-32	2819576
34	Randomized Controlled Trials as Topic/	164918
35	Randomized Controlled Trial/	602616
36	Random Allocation/	107038
37	Double Blind Method/	176559
38	Single Blind Method/	33030
39	Clinical Trial/	538977
40	Clinical Trial, Phase III.pt.	22146
41	Clinical Trial, Phase IV.pt.	2452
42	Controlled Clinical Trial.pt.	95446
43	Randomized Controlled Trial.pt.	602616
44	Multicenter Study.pt.	339543
45	Clinical Trial.pt.	538977
46	exp Clinical Trials as Topic/	385646
47	(clinical adj trial$).ab,ti.	491862
48	((single? or double? or treble? or triple?) adj (blind$3 or mask$3)).ab,ti.	200533
49	Placebos/	35933
50	Placebo?.ab,ti.	250703
51	randomly allocated.ab,ti.	37236
52	(allocated adj2 random*).ab,ti.	41065
53	or/34-52	1925975

54 9 and 33 and 53561

Embase (Elsevier)

#58. #10 AND #36 AND #56 AND [embase]/lim 1,460
#57. #10 AND #36 AND #56 1,477
#56. #37 OR #38 OR #39 OR #40 OR #41 OR #42 OR #43 OR #44 OR #45 OR #46 OR #47 OR #48 OR #49 OR #50 OR #51 OR #52 OR #53 OR #54 OR #55 2,983,764
#55. 'prospective study'/de 890,605
#54. placebo$:ab,ti 369,184
#53. ((treble OR triple) NEAR/1 blind*):ab,ti 2,080
#52. 'double blind*':ab,ti 247,731
#51. 'single blind*':ab,ti 32,000
#50. (random* NEAR/2 allocat*):ab,ti 55,265
#49. rct:ab,ti 55,218
#48. 'randomi?ed controlled trial$':ab,ti 327,907
#47. 'placebo'/de 411,398
#46. 'crossover procedure'/de 75,631
#45. 'double blind procedure'/de 212,308
#44. 'single blind procedure'/de 52,336
#43. 'randomization'/exp 99,050
#42. 'phase 4 clinical trial'/de 5,492
#41. 'phase 3 clinical trial'/de 70,182
#40. 'multicenter study'/de 375,932
#39. 'controlled clinical trial'/de 441,162
#38. 'randomized controlled trial'/de 791,446
#37. 'clinical trial'/de 1,086,127
#36. #11 OR #12 OR #13 OR #14 OR #15 OR #16 OR #17 OR #18 OR #19 OR #20 OR #21 OR #22 OR #23 OR #24 OR #25 OR #26 OR #27 OR #28 OR #29 OR #30 OR #31 OR #32 OR #33 OR #34 OR #35 2,266,185
#35. (immunologic NEAR/2 (factor$ OR receptor$)):ab,ti 1,761
#34. 'immunological antineoplastic agent'/exp 375,681
#33. 'immunologic factor'/de 4,180
#32. 'immunoglobulin receptor'/de 5,877
#31. durvalumab:ab,ti 3,838
#30. 'durvalumab'/de 10,658
#29. pembrolizumab:ab,ti 18,625
#28. 'pembrolizumab'/de 37,757
#27. camrelizumab:ab,ti 828
#26. 'camrelizumab'/de 2,083
#25. opdivo:ab,ti 165
#24. nivolumab:ab,ti 19,645
#23. 'nivolumab'/de 38,582

#22. 'pd l1':ab,ti	50,482
#21. 'pd 1':ab,ti	54,572
#20. "immune checkpoint inhibitor$":ab,ti	35,931
#19. 'immune checkpoint inhibitor'/de	26,291
#18. 'monoclonal antibod*':ab,ti	260,218
#17. 'monoclonal antibody'/de	229,300
#16. (immunosupressive NEAR/2 (therap* OR treatment$)):ab,ti	435
#15. 'immunosuppressive agent'/exp	1,437,170
#14. 'adoptive cell therap*':ab,ti	2,531
#13. immunization:ab,ti	125,344
#12. immunotherapy:ab,ti	177,327
#11. 'immunotherapy'/exp	319,193
#10. #1 OR #2 OR #3 OR #4 OR #5 OR #6 OR #7 OR #8 OR #9	75,822
#9. (ampullary NEAR/3 (cancer$ OR tumo?r OR neoplasm$)):ab,ti	1,334
#8. (ampulla NEAR/4 vater NEAR/4 cancer):ab,ti	270
#7. 'vater papilla carcinoma'/exp	3,340
#6. (gallbladder NEAR/3 (cancer$ OR tumo?r OR neoplasm$)):ab,ti	8,027
#5. cholangiocarcinoma$:ab,ti	28,852
#4. 'bile duct carcinoma'/exp	39,491
#3. ('bile duct' NEAR/3 (cancer$ OR tumo?r OR neoplas$)):ab,ti	3,496
#2. ('biliary tract' NEAR/3 (cancer$ OR tumo?r OR neoplasm$)):ab,ti	4,531
#1. 'biliary tract tumor'/exp	69,214

CENTRAL (Cochrane)

#1	MeSH descriptor: [Biliary Tract Neoplasms] explode all trees	654
#2	(("biliary tract" NEAR/3 (cancer? or tumor? or neoplasm?))):ti,ab,kw	801
#3	(("bile duct" NEAR/3 (cancer? or tumor? neoplasm?))):ti,ab,kw	821
#4	MeSH descriptor: [Cholangiocarcinoma] explode all trees	328
#5	(cholangiocarcinoma?):ti,ab,kw	957
#6	((gallbladder NEAR/3 (cancer? or tumor? or neoplasm?))):ti,ab,kw	434
#7	((ampullary NEAR/3 (cancer? or tumor? or neoplasm?))):ti,ab,kw	106
#8	((ampulla NEAR/3 vater NEAR/3 cancer)):ti,ab,kw	35
#9	{OR #1-#8}	2119

#10 MeSH descriptor: [Immunotherapy] explode all trees 11801

#11 (Immunotherapy):ti,ab,kw 12806

#12 (Immunization):ti,ab,kw 9498

#13 ("adoptive cell therapy" OR "adoptative cell therapies"):ti,ab,kw 26

#14 MeSH descriptor: [Immunosuppressive Agents] explode all trees 6169

#15 ("Immunosupressive therapy" OR "Immunosupressive therapies"):ti,ab,kw 17

#16 MeSH descriptor: [Antibodies, Monoclonal] explode all trees 18977

#17 ("monoclonal antibody" OR "monocloncal antibodies"):ti,ab,kw 10672

#18 MeSH descriptor: [Immune Checkpoint Inhibitors] explode all trees 203

#19 ("immune checkpoint inhibitor" OR "immune checkpoint inhibitors"):ti,ab,kw 1453

#20 (ICIs):ti,ab,kw 253

#21 (PD-1):ti,ab,kw 3227

#22 (PD-L1):ti,ab,kw 3914

#23 MeSH descriptor: [Nivolumab] this term only 788

#24 (opdivo):ti,ab,kw140

#25 (nivolumab):ti,ab,kw 2862

#26 (camrelizumab):ti,ab,kw 210

#27 (pembrolizumab):ti,ab,kw 2898

#28 (durvalumab):ti,ab,kw 1059

#29 MeSH descriptor: [Receptors, Immunologic] this term only 161

#30 MeSH descriptor: [Antineoplastic Agents, Immunological] this term only 642

#31 MeSH descriptor: [Immunologic Factors] this term only 1258

#32 (immunologic NEAR/2 (factor? or receptor?)) 1533

#33 {or #10-#32} 62403

#34 #9 and #33 in Trials 201

Web of Science (WOS). Meetings

Search: TS=("biliary tract" NEAR/3 (cancer$ or tumo$r or neoplasm$)) Results: 8491

Search: TS=("bile duct" NEAR/3 (cancer$ or tumo$r or neoplasm$)) Results: 25851

Search: TS=cholangiocarcinoma$ Results: 31586

Search: TS=(gallbladder NEAR/3 (cancer$ or tumo$r or neoplasm$)) Results: 15368

Search: TS=(ampullary NEAR/3 (cancer$ or tumo$r or neoplasm$)) Results: 1921

Search: TS=(ampulla NEAR/4 vater NEAR/4 cancer) Results: 458

Search: #1 OR #2 OR #3 OR #4 OR #5 OR #6 Results: 61995

Search: TS=Immuno* Results: 6737635

Search: TS=Immunization Results: 648805

Search: TS="adoptive cell therap*" Results: 2782

Search: TS=(Immunosupressive NEAR/2 (therap* or treatment$)) Results: 289

Search: TS="monoclonal antibod* " Results: 401285

Search: TS="immune checkpoint inhibitor$" Results: 35259

Search: TS=ICIs Results: 6570

Search: TS=PD-1 Results: 48077

Search: TS=PD-L1 Results: 42685

Search: TS=opdivo Results: 76

Search: TS=nivolumab Results: 25164

Search: TS=camrelizumab Results: 936

Search: TS=pembrolizumab Results: 20958

Search: TS=durvalumab Results: 3356

Search: TS=(immunologic NEAR/2 (factor$ or receptor$)) Results: 60214

Search: #8 OR #9 OR #10 OR #11 OR #12 OR #13 OR #14 OR #15 OR #16 OR #18 OR #19 OR #20 OR #17 OR #21 OR #22 Results: 7202900

Search: TS=("Randomi?ed Controlled Trial$") Results: 525822

Search: TS=(Clinical NEAR/2 Trial$) Results: 1007228

Search: TS=((single$ or double$ or treble$ or triple$) NEAR/0 (blind* or mask*)) Results: 487944

Search: TS=Placebo$ Results: 377760

Search: TS=(allocated NEAR/2 random*) Results: 52309

Search: #24 OR #25 OR #26 OR #27 OR #28 Results: 1808780

Search: #7 AND #23 AND #29 Results: 692

Search: #7 AND #23 AND #29 and Meeting (Document Types) Results: 84

Supplement S1

Search Results and Study Characteristics

The initial search identified 2306 publications. After excluding duplicates, 1987 publications remained. Of these, 1980 was discarded after reviewing the titles and abstracts. Following a full-text assessment, 5 publications were excluded, and 2 studies were included for data analysis (Process outlined in the PRISMA diagram in Figure S1). In case of multiple reports from the same trial, as previously described, the latest report meeting the inclusion and exclusion criteria was included.

Supplementary Figure S1. PRISMA Schema

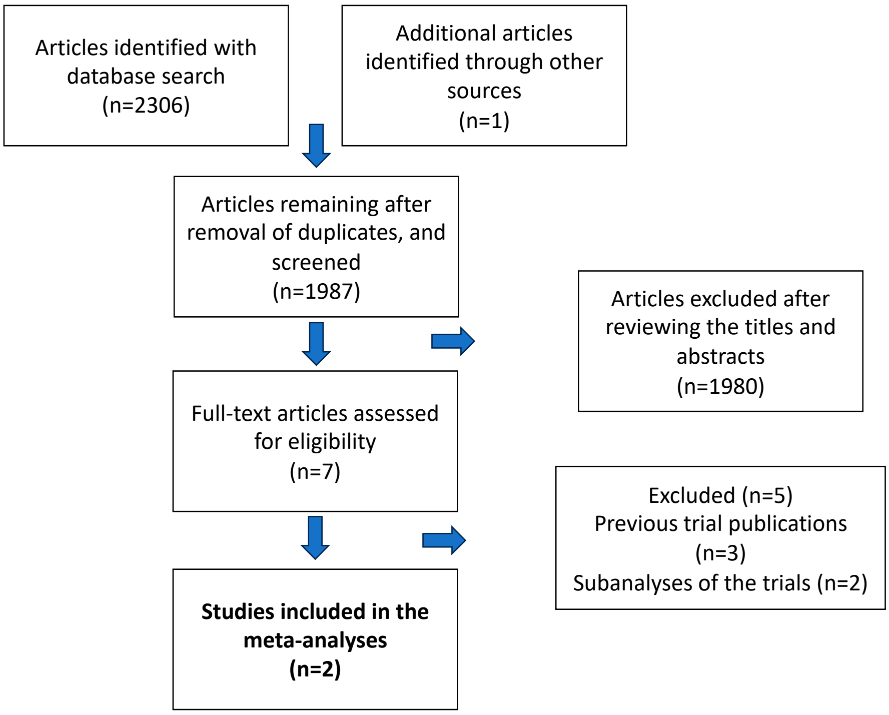

Supplement: Supplementary file 1 [file cancers-16-02077-s001.zip › Supplementary.pdf]
